# Effect of Peripheral Defocus on Axial Eye Growth and Modulation of Refractive Error in Hyperopes: Protocol for a Nonrandomized Clinical Trial

**DOI:** 10.2196/resprot.9320

**Published:** 2018-09-05

**Authors:** Ian G Beasley, Leon N Davies, Nicola S Logan

**Affiliations:** 1 Aston Optometry School Aston University Birmingham United Kingdom

**Keywords:** hyperopia, axial eye growth, amblyopia, hyperopic defocus, anisohyperopia

## Abstract

**Background:**

Hyperopia occurs due to insufficient ocular growth and a failure to emmetropize in childhood. In anisohyperopia, it is unclear why one eye may remain hyperopic while the fellow eye grows toward an emmetropic state. Animal studies have shown that manipulating peripheral defocus through optical means while simultaneously providing correct axial focus can either discourage or encourage axial eye growth to effectively treat myopia or hyperopia, respectively. Myopia progression and axial eye growth can be significantly reduced in children and adolescents through the use of multifocal contact lenses. These contact lenses correct distance central myopia while simultaneously imposing relative peripheral myopic defocus. The effect of correcting distance central hyperopia while simultaneously imposing relative peripheral hyperopic defocus is yet to be elucidated in humans.

**Objective:**

The objective of our study is to understand the natural progression of axial eye growth and refractive error in hyperopes and anisohyperopes and to establish whether axial eye growth and refractive error can be modified using multifocal contact lenses in hyperopes and anisohyperopes.

**Methods:**

There are 3 elements to the program of research. First, the natural progression of axial eye growth and refractive error will be measured in spectacle-wearing hyperopic and anisohyperopic subjects aged between 5 and <20 years. In other words, the natural growth of the eye will be followed without any intervention. Second, as a paired-eye control study, anisohyperopes aged between 8 and <16 years will be fitted with a center-near multifocal design contact lens in their more hyperopic eye and a single-vision contact lens in the fellow eye if required. The progression of axial eye growth and refractive error will be measured and compared. Third, subjects aged between 8 and <16 years with similar levels of hyperopia in each eye will be fitted with center-near multifocal design contact lenses in each eye; the progression of axial eye growth and refractive error in these subjects will be measured and compared with those of subjects in the natural progression study.

**Results:**

Recruitment commenced on 6 June 2016 and was completed on 8 April 2017. We estimate the data collection to be completed by April 2020.

**Conclusions:**

This trial will establish whether axial eye growth can be accelerated in children with hyperopia by imposing relative peripheral hyperopic defocus using center-near multifocal contact lenses.

**Trial Registration:**

ClinicalTrials.gov NCT02686879; https://clinicaltrials.gov/ct2/show/NCT02686879 (Archived by Webcite at http://www.webcitation.org/71o5p3fD2)

**Registered Report Identifier:**

RR1-10.2196/9320

## Introduction

### Background

Uncorrected hyperopia can cause blurring of distance vision and near vision and is a known risk factor for the development of strabismus and amblyopia [1]. There is a growing body of evidence that uncorrected hyperopia, in addition to visual consequences, may have a negative impact on educational attainment [2,3] and visuocognitive and visuomotor skills [4]. Hyperopia has received much less attention from research than myopia even though the impact of moderate to high levels of hyperopia, especially in one eye (anisohyperopia), can lead to amblyopia if not fully corrected at a young age [5]. Hyperopia occurs as a consequence of insufficient ocular growth and a failure to emmetropize in childhood, with the majority of hyperopic refractive errors resulting from an eye that is too short for its refractive power [6]. In anisohyperopia, it is unclear why one eye may remain hyperopic while the fellow eye grows toward an emmetropic state. Myopic eyes typically exhibit relative hyperopic blur in the periphery [7,8], which is thought to be a precursor of the development of myopia [9]. Studies on animals have suggested that manipulating peripheral defocus through optical means while simultaneously providing correct axial focus can either discourage or encourage axial eye growth to effectively treat myopia or hyperopia, respectively [10].

It has been established that myopia progression and axial eye growth can be significantly reduced in children and adolescents through the use of bifocal or dual-focus contact lenses [11-13]. These contact lenses are designed to correct distance central myopia while simultaneously imposing relative peripheral myopic defocus. This intervention relies on active accommodation, and the myopia control studies have shown that children accommodate normally with multifocal contact lenses [11]. In contrast to the myopic eye, the hyperopic eye tends to exhibit relative peripheral myopia [8].

This study explores the use of center-near multifocal design contact lenses to correct distance central hyperopia while simultaneously imposing relative peripheral hyperopic defocus. The aim is to determine, for the first time, whether axial eye growth can be accelerated in children with hyperopia and anisohyperopia to reduce the refractive error and improve visual outcome.

### Study Objectives

The objectives of our study are to understand the natural progression of axial eye growth and refractive error in hyperopes and anisohyperopes, and to establish if axial eye growth and refractive error can be modified using center-near multifocal design contact lenses in hyperopes and anisohyperopes to improve visual outcome.

## Methods

### Study Design

There will be 3 elements to the proposed program of research:

1. *Natural progression*: Refractive error and axial eye growth will be followed over a 3-year period in hyperopic and anisohyperopic participants aged between 5 and <20 years to gain an understanding of the natural progression of these parameters in the specified cohort. This arm of the study does not involve an intervention.
2. *Hyperopic intervention*: For this part of the study, refractive error and axial eye growth will be followed in participants aged between 8 and <16 years over a 6-month period, after which the participants will wear bifocal soft contact lenses daily for 2 years. The intervention will then be withdrawn before the final data collection point 6 months later. The center-near design contact lenses will provide clear central vision at both distance and near, thus, exposing the retina to peripheral hyperopic defocus from the distance zone.
3. *Anisohyperopic intervention:* Anisometropes represent a unique example of ocular development, where the two eyes of an individual, with an identical genetic background and seemingly subject to identical environmental influences, can grow asymmetrically to produce significantly different refractive errors. Axial eye growth and refractive error will be followed in participants aged between 8 and <16 years over a 6-month period before being fitted with a center-near design bifocal soft contact lens in their more hyperopic eye, while the fellow eye will be fitted with a single-vision contact lens if required. The contact lenses will be worn daily for 2 years. The intervention will then be withdrawn before the final data collection point 6 months later. If and when the refractive error in the more hyperopic eye has reached a level similar to that in the less hyperopic eye (<0.25 diopter [D] interocular difference), both eyes will be fitted, if necessary, with bifocal center-near design contact lenses in line with the protocol for participants in the hyperopic intervention group.

For the study, healthy hyperopic children and adolescents will be recruited via the researchers’ optometry practices and Aston University's Eye Clinic. A database search will be performed by the immediate research team to identify potential participants who meet the criteria for inclusion; these individuals will be contacted via post with information regarding the study and how to participate. A poster will also be displayed at both research venues as a means of recruiting other potential participants. Contact details will be stated on the poster to enable anyone interested in joining the study to contact the researcher for further details.

All participants, including parent(s) or guardian(s), expressing an interest in joining the study or wishing to recruit their child into the study will be provided detailed information regarding the study and will have the opportunity to ask questions before completing the consent form declaration.

Allocation to the contact lens-wearing and the natural progression arms of the study will not be randomized. Individuals who are willing and able to use contact lenses will be given the opportunity to be included in the contact lens arm of the study; those who do not want to wear, are unable to handle, or unsuitable for contact lenses will be given the opportunity to participate in the natural progression arm of the study.

After obtaining informed consent, all participants will undergo a number of visual assessments including a background questionnaire, axial length measures, subjective refraction, accommodative lag, amplitude of accommodation, stereopsis, vision and visual acuity, postcycloplegic objective refraction, postcycloplegic peripheral refraction at 30**°** temporally and nasally, and 20**°** superiorly and inferiorly, and pupil size.

For the natural progression study, the normal growth of the eyes will be followed at 7 visits over 3 years without any intervention. A topical cycloplegic (cyclopentolate hydrochloride 1%) will be used on visits 1, 2, 4, 6, and 7 (Table 1).

Participants in the intervention arms of the study will also have the normal growth and visual characteristics of the eyes followed at 7 visits over 3 years. In addition, they will have their suitability for contact lens wear determined at the second visit, 6 months after the first visit, which will require instructions on contact lens wear and follow-up appointments. In total, for participants in the intervention arms of the study, there will be 9 scheduled visits, with a cycloplegic used on visits 1, 2, 6, 8, and 9 (Table 2). There may be unscheduled visits if any problems arise as a result of contact lens wear. The participants, with parental support where necessary, will be instructed on how to insert, remove, and care for their contact lenses.

**Table 1:** Procedures for participants in the natural progression arm of the study.

Procedure	Visit 1 (month 1)	Visit 2 (month 6)	Visit 3 (month 12)	Visit 4 (month 18)	Visit 5 (month 24)	Visit 6 (month 30)	Visit 7 (month 36)
Informed consent	✓	✓	✓	✓	✓	✓	✓
Questionnaire	✓	✓	✓	✓	✓	✓	✓
Axial length	✓	✓	✓	✓	✓	✓	✓
Subjective refraction	✓	✓	✓	✓	✓	✓	✓
Accommodative lag	✓	✓	✓	✓	✓	✓	✓
Amplitude of accommodation	✓	✓	✓	✓	✓	✓	✓
Stereopsis	✓	✓	✓	✓	✓	✓	✓
Vision and visual acuity	✓	✓	✓	✓	✓	✓	✓
Postcycloplegic refraction	✓	✓		✓		✓	✓
Peripheral refraction	✓	✓		✓		✓	✓

**Table 2:** Procedures for participants in the intervention arms of the study.

Procedure	Visit 1 (month 1)	Visit 2 (month 6)	Visit 3 (month 7)	Visit 4 (month 8)	Visit 5 (month 12)	Visit 6 (month 18)	Visit 7 (month 24)	Visit 8 (month 30)	Visit 9 (month 36)
Informed consent	✓	✓			✓	✓	✓	✓	✓
Questionnaire	✓	✓			✓	✓	✓	✓	✓
Axial length	✓	✓			✓	✓	✓	✓	✓
Subjective refraction	✓	✓			✓	✓	✓	✓	✓
Accommodative lag	✓	✓			✓	✓	✓	✓	✓
Amplitude of accommodation	✓	✓			✓	✓	✓	✓	✓
Stereopsis	✓	✓			✓	✓	✓	✓	✓
Vision and visual acuity	✓	✓			✓	✓	✓	✓	✓
Postcycloplegic refraction	✓	✓				✓		✓	✓
Peripheral refraction	✓	✓				✓		✓	✓
Pupil size					✓				
Contact lens fitting		✓							
Contact lens aftercare			✓	✓	✓	✓	✓	✓	

For the anisohyperopes, the more hyperopic eye will be fitted with a commercially available soft multifocal contact lens and the less hyperopic eye will be fitted with a soft single-vision contact lens, if required. Participants with similar levels of hyperopia in each eye will be fitted with commercially available soft multifocal contact lenses in both eyes.

For anisohyperopic subjects, if the level of hyperopia in the more hyperopic eye reduces to a level which is similar to that of the less hyperopic eye, both eyes will be fitted with multifocal contact lenses if sufficient hyperopia remains, in line with the inclusion criteria for the second arm of the study.

For nonanisohyperopic subjects, the intervention will be stopped when the refractive error has reached a mean spherical error of +0.50 D. At the end of an intervention period of 24 months, contact lens wear will cease and the participants will be assessed for the final time after an interval of 6 months.

### Inclusion Criteria

To be included in the study, the participants had to be aged between 5 and <20 years at the initial examination for the natural progression study and between 8 and <16 years at the initial examination for the intervention study. Furthermore, the parents must have read, understood, and signed the informed consent form, and the participants must have read, understood, and signed the assent form. The participants in the intervention groups had to agree to wear the prescribed contact lenses for a minimum of 10 hours per day, at least 6 days per week, for the 2-year duration of the intervention period and be in good general health with no contraindications to contact lens wear. Additionally, the following criteria had to be met: maximum manifest spherical refractive error of +6.00 D, maximum manifest cylindrical refractive error of −1.00 D, minimum anisometropia of >1.00 D in the anisohyperopic group (mean spherical error), maximum anisometropia of 1.00 D in the nonanisohyperopic group (mean spherical error), and minimum mean spherical refractive error of +2.00 D in the more hyperopic eye. Furthermore, participants had to be competent at handling contact lenses and understand the instructions provided to ensure safe wear.

### Exclusion Criteria

The exclusion criteria were as follows: previous contact lens wear, participation in another clinical study, regular use of medication to treat ocular conditions, current use of systemic medication that may have an impact upon successful contact lens wear or vision, known ocular or systemic disease, findings identified during contact lens assessment that would preclude contact lens wear, and not being able to provide informed consent without the aid of an interpreter, as no funding was available for the provision of interpreter facilities.

### Ethical Approval

The study was granted ethical approval by the National Health Service Research Ethics Committee on May 26, 2016, and by Aston University Research Ethics Committee on June 2, 2016. The trial is registered at ClinicalTrials.gov (NCT02686879).

### Sample Size

Sample size has been calculated using G*Power (version 3.1.9; Franz Faul, Universität Kiel, Germany) for a significance level of alpha=.05 at 80% power while also allowing for attrition.

### Statistical Analyses

All data will be analyzed using the commercially available software SPSS version 23 (IBM, NY, United States) using a repeated-measures mixed analysis of variance design with 1 between-subject factor and 1 within-subject factor. Principal outcomes measures are: change in axial eye growth and change in refractive error.

## Results

Recruitment commenced on June 6, 2016, and was completed on April 8, 2017. We estimate the data collection to be completed by April 2020.

## Discussion

The prevalence of myopia is increasing at an alarming rate in many parts of the world [14]. Given the association between myopia and increased risk of ocular comorbidity later in life due to excessive axial eye growth [15], the need to arrest myopia progression during childhood is clear.

There is now compelling evidence that axial eye growth and the subsequent myopia progression can be slowed through a range of interventions, including the use of multifocal contact lenses [11-13]. However, despite the lifelong burden and visual consequences of hyperopia commencing in early childhood, there is a paucity of evidence in relation to modulating axial eye growth in this cohort.

If it is possible to slow axial eye growth in myopes by manipulating the peripheral retinal image shell, encouraging axial eye growth in hyperopes using the same rationale is plausible, and this notion is supported by the literature on animal research [10]. Further, peripheral refraction measures differ as a result of retinal shape, with myopes typically exhibiting relative peripheral hyperopia and hyperopes tending to be relatively myopic in the periphery [8]. These characteristics support the proposal to impose relative peripheral hyperopic defocus aimed at stimulating axial eye growth in hyperopes. Indeed, the progression of axial myopia in children is associated with hyperopic relative peripheral defocus [16]. In addition to the primary outcome measures, changes in the relative peripheral refraction as a result of the intervention will be elucidated.

The clinical trial outlined here will determine, for the first time, whether axial eye growth and refractive error can be accelerated in hyperopic children using multifocal contact lenses. The outcome could have far-reaching implications for the visual prognosis of children with hyperopia.

## Appendix

Multimedia Appendix 1 Peer-reviewer report #1 from the College of Optometrists, London, UK (funder).

Multimedia Appendix 2 Peer-reviewer report #2 from the College of Optometrists, London, UK (funder).

## Abbreviations

D: diopter

## References

[1] Colburn JD, Morrison DG, Estes RL, Li C, Lu P, Donahue SP (2010). Longitudinal follow-up of hypermetropic children identified during preschool vision screening. J AAPOS.

[2] Williams WR, Latif AHA, Hannington L, Watkins DR (2005). Hyperopia and educational attainment in a primary school cohort. Arch Dis Child.

[3] Narayanasamy S, Vincent SJ, Sampson GP, Wood JM (2014). Simulated hyperopic anisometropia and reading, visual information processing, and reading-related eye movement performance in children. Invest Ophthalmol Vis Sci.

[4] Atkinson J, Braddick O, Nardini M, Anker S (2007). Infant hyperopia: detection, distribution, changes and correlates-outcomes from the cambridge infant screening programs. Optom Vis Sci.

[5] Kulp MT, Ying G, Huang J, Maguire M, Quinn G, Ciner EB, Cyert LA, Orel-Bixler DA, Moore BD, Study Group VIP (2014). Associations between hyperopia and other vision and refractive error characteristics. Optom Vis Sci.

[6] Strang NC, Schmid KL, Carney LG (1998). Hyperopia is predominantly axial in nature. Curr Eye Res.

[7] Ehsaei A, Mallen EAH, Chisholm CM, Pacey IE (2011). Cross-sectional sample of peripheral refraction in four meridians in myopes and emmetropes. Invest Ophthalmol Vis Sci.

[8] Mutti DO, Sholtz RI, Friedman NE, Zadnik K (2000). Peripheral refraction and ocular shape in children. Invest Ophthalmol Vis Sci.

[9] Mutti DO, Hayes JR, Mitchell GL, Jones LA, Moeschberger ML, Cotter SA, Kleinstein RN, Manny RE, Twelker JD, Zadnik K, CLEERE Study Group (2007). Refractive error, axial length, and relative peripheral refractive error before and after the onset of myopia. Invest Ophthalmol Vis Sci.

[10] Benavente-Pérez A, Nour A, Troilo D (2014). Axial eye growth and refractive error development can be modified by exposing the peripheral retina to relative myopic or hyperopic defocus. Invest Ophthalmol Vis Sci.

[11] Anstice NS, Phillips JR (2011). Effect of dual-focus soft contact lens wear on axial myopia progression in children. Ophthalmology.

[12] Lam CSY, Tang WC, Tse DY, Tang YY, To CH (2014). Defocus Incorporated Soft Contact (DISC) lens slows myopia progression in Hong Kong Chinese schoolchildren: a 2-year randomised clinical trial. Br J Ophthalmol.

[13] Walline JJ, Greiner KL, McVey ME, Jones-Jordan LA (2013). Multifocal contact lens myopia control. Optom Vis Sci.

[14] Holden BA, Fricke TR, Wilson DA, Jong M, Naidoo KS, Sankaridurg P, Wong TY, Naduvilath TJ, Resnikoff S (2016). Global Prevalence of Myopia and High Myopia and Temporal Trends from 2000 through 2050. Ophthalmology.

[15] Flitcroft DI (2012). The complex interactions of retinal, optical and environmental factors in myopia aetiology. Prog Retin Eye Res.

[16] Yamaguchi T, Ohnuma K, Konomi K, Satake Y, Shimazaki J, Negishi K (2013). Peripheral optical quality and myopia progression in children. Graefes Arch Clin Exp Ophthalmol.

