# Supplementary material for: Effect of Peripheral Defocus on Axial Eye Growth and Modulation of Refractive Error in Hyperopes: Protocol for a Nonrandomized Clinical Trial
Source: JMIR Res Protoc. 2018 Sep 5;7(9):e173. doi: 10.2196/resprot.9320 (PMC6231719; doi:10.2196/resprot.9320)
Supplement: Multimedia Appendix 1 [file resprot_v7i9e173_app1.pdf]

THE COLLEGE OF OPTOMETRISTS

Postgraduate Scholarships 2015 - Review Form

APPLICATION DETAILS

**Title of study:**	The effect of peripheral defocus on axial growth and modulation of refractive error in hyperopes
**Dept. making application:**	Optometry and Vision Science – ASTON University
**Proposed Supervisor:**	Dr. Nicola Logan

REVIEW CRITERIA

A. Is the question important and relevant to ophthalmic optics and related subjects?

Yes of course, despite myopia has attracted most of the attention in the recent years, hyperopia is still the main refractive error in may age groups and usually causes visual efficiency problems.
The project has also the potential to serve presbyopia correction throuht induction of low values of myopia in the non-dominant eye and would have also the potential to be applied in older children and young adults where other treatment options are not usually considered to low or moderate hyperopes.

**dScore**	Of no importance					Very important
(please circle)	**0**	**1**	**2**	**3**	**4**	**(5)**

B. Is the research original?

The research to be conducted is original and addresses a relevant topic. Beyond the desirable applications in the study of hyperopia development, the study could bring new light into the emmetropization processes of the human eye. Although myopia is now a hot topic and the main investigator in this study has a strong background in the field, this study would indirectly contribute originally to help to explain the mechanisms of eye growth.

**Score**	Not original					Very original
(please circle)	**0**	**1**	**2**	**3**	**4**	**(5)**

C. Is the study appropriately and well designed?

The study is appropriately designed. The methods are also well described with the sample size well justified. The ethical issues would be very important considering the nature of the intervention as the researchers aim to “stimulate” eye growth which is very different from what most researchers in the field of myopia aim to do, to slow down eye growth. The authors clearly state that they would find ethical approval.
The authors will follow the intervention for 10 months, which might be not enough time to consolidate the results, but this is indeed an unknown information. Perhaps a pilot study in some case reports could be conducted in advance prior to enrol all the participants. This would allow to estimate the minimum time to obtain a growth response from the eye.
Another consideration is to conduct this study with older children. At the age of 8 to 12, the children could still be in the emmetropization process towards Emmetropia. To stimulate their eye growth could be a trigger for faster myopization. The authors should consider mechanisms to ensure that their subjects are stable hyperopes, instead of emmetropizing hyperopes.

**Score**	Poorly designed					Very well designed
(please circle)	**0**	**1**	**2**	**3**	**4** (circled)	**5**

D. How do you rate the quality of the application overall?

The application is of very good quality, with a good description of the background and potential benefits of the study. However, further details could be provided in the description of the different studies planned. Despite this, the information is enough to evaluate the scientific potential of the project.

**Score**	Very poor quality					Very good quality
(please circle)	**0**	**1**	**2**	**3**	**4** (circled)	**5**

E. Is the applicant appropriate for training and development of the Scholar in research? What previous experience has s/he had in training individuals for research? Include relevant details such as previous PhD's supervised (names of students, dates registered and dates completed), publications in the area of research being proposed.

YES, the principal investigator has a track of 7 students completing their PhD under her supervision (5 as principal supervisor). This has been accomplished in a very short period of time from 2007 till 2015, at a rate of 1 PhD thesis/year, which is a very high rate in the field. The conclusion is that the principal investigator is strongly committed with the post-graduate supervision and has a highly efficient activity in this field.

**Score**	Not appropriate					Very appropriate
(please circle)	**0**	**1**	**2**	**3**	**4**	**5**

F. Does the Department have the appropriate support in place (if known)?

Yes, this is a very well equipped lab with staff involved in emmetropization studies for several years now.
The principal investigator has a long track of research and high impact publications in the field of refractive error development and ocular biometry.

**Score**	Poor support					Very good support
(please circle)	**0**	**1**	**2**	**3**	**4**	**5**

REVIEWER DETAILS

Name:

Signature:

SUBMISSION OF COMPLETED REVIEW FORM

The completed form should be returned **no later than Thursday 2 April** to:
researchteam@college-optometrists.org
