# Supplementary material for: Effect of Peripheral Defocus on Axial Eye Growth and Modulation of Refractive Error in Hyperopes: Protocol for a Nonrandomized Clinical Trial
Source: JMIR Res Protoc. 2018 Sep 5;7(9):e173. doi: 10.2196/resprot.9320 (PMC6231719; doi:10.2196/resprot.9320)
Supplement: Multimedia Appendix 2 [file resprot_v7i9e173_app2.pdf]

Nicola Logan Postgraduate Scholarship – peer review feedback

Review 1:

The PhD programme proposed investigates the efficacy of bifocal/multifocal lenses for the correction of hyperopia. This is an area of research that has received limited attention thus far despite the huge clinical impact it is likely to have. Determining effective methods of arresting hyperopia would have a profound effect on how clinicians manage this type of refractive error and as such the study proposal is highly relevant to ophthalmic optics and vision sciences. Furthermore the proposed work may potentially provide further understanding of the mechanism by which refractive changes occur in the developing eye.

Much of the evidence on manipulating refractive error via contact lenses inducing peripheral defocus has been focused on myopia; to the reviewer's knowledge this study would be the first interventional study to assess the effectiveness of using multifocal contact lenses to modulate hyperopic refractive error. The research project proposed is highly original yet based on a body of literature that suggests that peripheral defocus governs ocular growth.

The study proposed has clearly been well thought out. Refractive error research is notoriously difficult due to the significant number of genetic and environmental confounding variables that can affect the outcome. Using the subjects as their own control (i.e. the most hyperopic eye will be given the intervention contact lens and the fellow eye the standard lens) overcomes this issue particularly well. The time scale for the study and the proposed outcome measures are appropriate for this type of research. It might be worth the investigators examining the effectiveness of the near addition for the multifocal contact lens prior to the trial commencing as there is some evidence to suggest that the quality of vision generated by the near addition of these types of lenses is not always consistent.

The application being reviewed is extremely well designed and succinctly identifies the research question and study proposal. The literature discussed outlines the basis of the research question and provides a clear reasoning for applying current knowledge of peripheral defocus via myopia control lenses to hyperopic eyes. The proposal is feasible in the time frame suggested and the outcome measures are relevant and achievable. This area of research could have significant implications for our understanding of refractive error development but also provides a means of investigating a potential strategy for correcting and managing hyperopic refractive error. The investigators have presented a clear plan of study including details on how subjects will be screened, recruited and monitored over the two-year study period. Furthermore to monitor the stability of any potential refractive error change the subjects will be followed for a further 6 months after finishing the study. In view of the principal investigator's research background this type of study is ideally suited.
